# Humoral responses to wild type and ancient BA.1 SARS-CoV-2 variant after heterologous priming vaccination with ChAdOx1 nCoV-19 and BNT162b2 booster dose

**DOI:** 10.1007/s10238-023-01276-x

**Published:** 2024-01-20

**Authors:** Giuseppina Sanna, Alessandra Marongiu, Davide Firinu, Cristina Piras, Vanessa Palmas, Massimiliano Galdiero, Luigi Atzori, Paola Caria, Marcello Campagna, Andrea Perra, Giulia Costanzo, Ferdinando Coghe, Roberto Littera, Luchino Chessa, Aldo Manzin

**Affiliations:** 1 Department of Biomedical Sciences, University of Cagliari, Cittadella Universitaria, 09042 Monserrato, Cagliari, Italy; 2 Department of Medical Sciences and Public Health, University of Cagliari, Cittadella Universitaria, 09042 Monserrato, Cagliari, Italy; 3 Clinical Metabolomics Unit, Department of Biomedical Sciences, University of Cagliari, Cittadella Universitaria, 09042 Monserrato, Cagliari, Italy; 4 Department of Experimental Medicine, University of Campania Luigi Vanvitelli, 80138 Naples, Italy; 5 Unit of Oncology and Molecular Pathology, Department of Biomedical Sciences, University of Cagliari, Cittadella Universitaria, 09042 Monserrato, Cagliari, Italy; 6 Laboratory Clinical Chemical Analysis and Microbiology, University Hospital of Cagliari, 09042 Monserrato, Italy; 7 Medical Genetics, Department of Medical Sciences and Public Health, University of Cagliari, 09100 Cagliari, Italy

**Keywords:** COVID-19, Omicron BA.1, BNT162b2 vaccine, ChAdOx1-S vaccine, Booster, Anti-S-IgG, Neutralizing antibodies

## Abstract

Several countries have recommended a booster dose of Pfizer BNT162b2 vaccine for subjects under the age of 60, who have already received the first dose of ChAdOx1. This is due to several ChAdOx1 vaccine-associated adverse vascular events and thrombocytopenia. Neutralization assay and quantitative IgG anti-SARS-CoV-2 Spike antibody (anti-S-IgG) were conducted to investigate the long-term responses to vaccine treatment in a cohort of Sardinian participants, who have received heterologous Prime–Boost Vaccination via ChAdOx1 vector vaccine and a booster dose via BNT162b2. The obtained results were compared with those of a cohort of healthcare workers (HCW) who received homologous BNT162b2 (BNT/BNT/BNT) vaccination. One month (T2) and five months after the second and before the third dose (T3), anti-spike antibody or neutralizing titers in the subjects vaccinated with ChAdOx1-S/BNT162b2 were significantly higher than those who experienced the ChAdOx1-S/ChAdOx1-S or BNT162b2/BNT162b2 schedule. These results suggest that a ChAdOx1-S/BNT162b2 regimen provides a more robust antibody response than either of the homologous regimens. However, the anti-spike antibodies or neutralizing titers after the third injection (mRNA vaccine) of ChAdOx1-S as a second dose and BNT162b2 were not statistically different. Homologous and heterologous vaccination provided a strong antibody response. Neutralizing activities were also described against the Omicron BA.1 variant in a sub-group (40) representative of the three vaccination regimens among our cohort.

## Introduction

Since the advent of the SARS-CoV-2 pandemic in September 2023, over 770 million confirmed cases and 6,95 million deaths have been reported globally (WHO data) [1].

Food Drug Administration (FDA) announced the first approval of a COVID-19 vaccine in August 2021 [2].

The BNT162b2, marketed as Comirnaty, also known as the Pfizer-BioNTech mRNA COVID-19 Vaccine, and administered as a two-dose primary series.

After that, multiple successful vaccines against the coronavirus SARS-CoV-2 have been developed. Among the authorized ones, the ChAdOx1 nCoV-19 adenovirus-based vector vaccine (ChAdOx1, hereafter referred to as ChAd) [3] and the two mRNA vaccines (BNT162b2 and mRNA-1273) [4] have been employed the most.

However, ChAdOx1 vaccine-associated adverse vascular events and mainly the occurrence of Vaccine-induced immune thrombotic thrombocytopenia (VITT) have limited some countries' employment of the aforementioned vaccines [5–7].

The occurrence of said rare adverse events has led to revised recommendations in support of an ulterior vaccination for all subjects who have received the first ChAdOx1 dose.

A complete vaccination via vector vaccine was recommended for 60-year-old subjects, whereas heterologous boosting via mRNA vaccine was recommended for individuals younger than 60 (years) [8].

Improving knowledge of SARS-CoV-2 Receptor Binding Domain (RBD) antibody kinetics after vaccination with different vaccine types and regimens is crucial for developing approaches able to increase the vaccine coverage and the impact within populations, also in the context of emerging variants such as Omicron lineage. In many countries including Italy, a large part of the population did not undergo additional booster of COVID-19 vaccines beyond the third dose, thus being exposed to the infection of the Omicron lineage [9, 10].

Aiming to explore the immunogenicity of the heterologous prime-boost vaccination, we have employed the prospective cohort of subjects naive to SARS-CoV-2 enrolled in our COVID-19 research project CORIMUN [11] and verified the responses to vaccine combinations such as that with heterologous priming with ChAdOx1 (ChAd) vector vaccine, followed by boosting with BNT162b2, hereafter referred to as BNT, (ChAd/BNT/BNT; *n* = 23) and ChAd/ChAd/BNT, *n* = 23), or homologous regimen BNT/BNT/BNT (*n* = 20) as a control group.

We have evaluated a total of 228 sera for neutralization titers and antibody responses to SARS-CoV-2 spike protein at 4 and 20 weeks after the second dose and 4 weeks after the third dose, with a homologous or heterologous vaccine combination. Furthermore, there is limited knowledge about humoral response against the variants of concern (VOCs), especially against the Omicron BA.1 variant, the most prevalent in Sardinia at the time of this study. Thus, we have then further investigated the neutralization competence against the Omicron BA.1 variant, after a completed three-dose vaccination schedule, in a sample (*n* = *40*) of subjects among our cohort treated with the three vaccine combinations.

In the same cohort, we have analyzed and compared the neutralizing activity against the SARS-CoV-2 *wt* and Omicron BA.1 variant after the BNT162b2 third dose in a subgroup of subjects (16 included in the BNT/BNT group and 14 in the homologous ChAd/ChAd and heterologous ChAd/BNT group) that have tested positive for SARS-CoV-2.

## Materials and methods

### Cohort participants

**Fig. 1:**
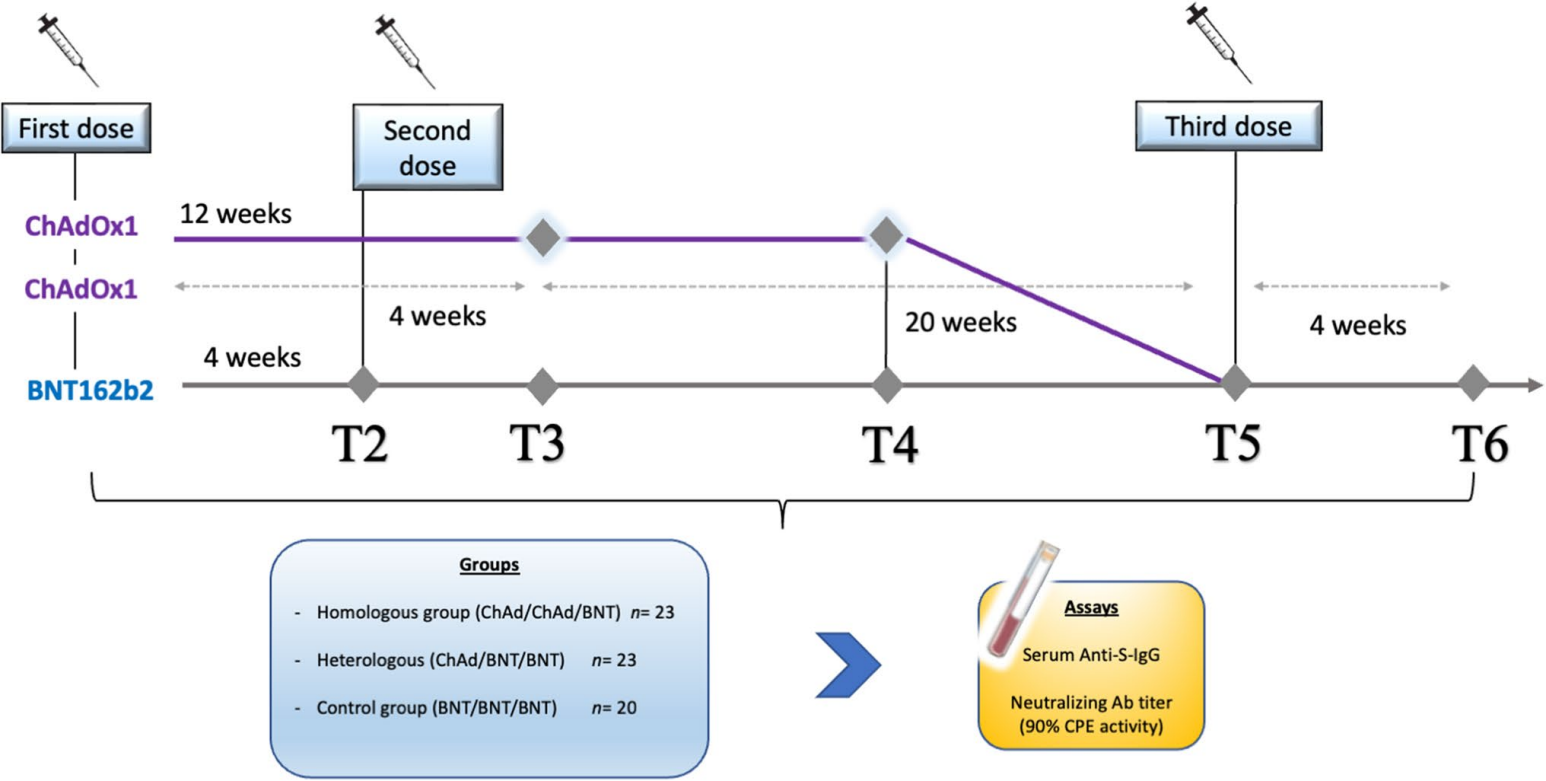
Time points of the study

The present observational study enrolled a cohort of participants from Regione Sardegna (*n* = 66). All subjects were assorted for age and gender and limited to immunocompetent participants. The group included healthcare workers, post-graduate medical trainees, and researchers at the University of Cagliari, who had undergone either a heterologous COVID-19 vaccination (ChAdOx1 nCoV-19 as prime and BNT162b2 mRNA as a booster (ChAd/BNT), or homologous ChAdOx1 nCoV-19 (ChAd/ChAd) or BNT162b2 (BNT/BNT) vaccination regimens. According to the guidelines, booster vaccination took place approximately 21 days after BNT prime and 3 months after ChAd prime (Fig. 1). Subjects were recruited and enrolled in the study protocol at the Teaching Hospital of Cagliari University. After having provided written informed consent, we have collected peripheral blood samples by venipuncture. Cohorts’ member characteristics are shown in Table 1.

**Table 1:** General characteristics of cohort participants

	Heterologous vaccine (*n* = 23)	Homologous vaccine (*n* = 23)	HCW control group (*n* = 20)
Age in years (median (IQR))	39	52.4	48.7
Female (%)	84	73	64.7
Currently smoker (*n* (%))	10	14	11.7
Presence of comorbidity (*n* (%))	0	0	0
Weeks between vaccine doses, median (IQR)	11.5 (0.3)	12 (0.2)	3 (0)

Heterologous: first dose of ChAdOx1 nCoV-19, second dose BNT162b2. Homologous: two doses of ChAdOx1. HCW: two doses of BNT162b2 mRNA

### Sample collection

The samples of peripheral blood (10 mL) were obtained through venipuncture and named T2 4 weeks after the booster dose (full vaccination course), T3before the third dose, and T5 4 weeks after the third dose. The serum (a total of 228 samples) was obtained by centrifugation (3000 rpm × for 15 min) within three hours from collection and aliquots were stored at − 80 °C until use.

### Serology

SARS-CoV-2 S-RBD IgG CLIA for the in vitro quantitative determination of antibodies (IgG) against S-RBD in human serum was performed on Maglumi 800 analyzer (SNIBE—Shenzhen New Industries Biomedical Engineering Co., Ltd, Shenzhen, Cina). The results were expressed as arbitrary units (AU) per milliliter and, according to the manufacturer's claims, a test result ≥ 1.10 AU/mL was considered positive.

### Neutralization assay (NA)

NA was performed in the Biosafety Level 3 (BSL-3) laboratory (Section of Microbiology and Virology, Cittadella Universitaria di Monserrato) as previously described [12]. Serum samples were diluted (1:10; 1:40; 1:160; 1:640) in triplicates and mixed with 100 TCID_50_ of SARS-CoV-2 virus (clinical isolate, strain VR PV10734, kindly donated by the Lazzaro Spallanzani Hospital of Rome, Italy), and BA.1 variant (clinical isolate, strain EPI_ISL_13398512) at 37 °C, serum/virus mixes were transferred to 96-wells containing 5 × 10^5^/mL adherent Vero E6 and Vero 76 (ATCC, Manassas, Virginia, United States) cells, respectively, seeded the day before in.

Plates were incubated at 37 °C for 72 h or 96 (BA.1 variant) prior to evaluation of CPE via microscope and were then fixed and stained with Gram’s crystal violet solution. The neutralization percentage of the individual dilutions was calculated by setting the mean OD595 of the serum control equal to 100%. Virus dilution used for infection was titrated in each experiment. Cell growth and serum controls were run in each experiment. Neutralization titers of serum samples were determined by the highest serum dilution protecting 90% of the infected wells [13].

### Statistical analysis

GraphPad Prism software (version 7.01, GraphPad Software, Inc., San Diego, CA, USA) was used to perform the univariate statistical analysis.

## Results

Among the vaccinated participants (*n* = 66) still naïve to SARS-CoV-2 infection, 20 subjects were included in the BNT/BNT group, 23 in the ChAd/ChAd group, and 23 in the ChAd/BNT group. Only Caucasian individuals were available for this study. Analyzed subjects were homogeneous in regard to age, gender, and smoking habits.

97% (64/66) of participants involved in the vaccination campaign have developed antibody responses (positively defined by > 1 AU/mL) at designed times T2, T3, and T5.

### Anti-S-RBD antibody levels and neutralization titers 4 weeks (T2) after the second dose

IgG anti-S-RBD values of cohort participants evaluated at T2 (after the second dose) seroconverted, exhibiting results above the cut-off value of 1 AU/mL.

Table 2 reports the median IgG anti-S-RBD levels and IQR for each studied group (BNT/BNT, ChAd/ChAd, ChAd/BNT).

**Table 2:** IgG anti-S-RBD median values of cohort participants evaluated at T2 (4 weeks after the second dose), T3 (20 weeks after the second dose), and T5 (4 weeks after the third dose)

IgG anti-S-RBD AU/ml	1st Quartile	Median	3rd Quartile	IQR (Interquartile Range)	Comparison versus ChAd/BNT/BNT *p-*Value*	Comparison versus BNT/BNT/BNT *p-*Value*
T2						
ChAd/BNT/BNT (*n* = 20)	193.4	475.7	873.3	679.9	–	0.011
ChAd/ChAd/BNT (*n* = 20)	11.95	50.16	94.4	82.45	< 0.0001	0.101
BNT/BNT/BNT (*n* = 20)	63.2	111.8	151.3	88.1	0.011	–
T3						
ChAd/BNT/BNT	14.96	42.91	92.98	78.02	–	0.102
ChAd/ChAd/BNT	2.05	5.34	16.55	14.5	0.0006	0.387
BNT/BNT/BNT	5.84	13	33.07	27.23	0.102	–
T5						
ChAd/BNT/BNT	450.7	775.3	1022	571.3	–	0.170
ChAd/ChAd/BNT	413.7	849	1022	608.3	> 0.99	0.177
BNT/BNT/BNT	203.1	410.5	980	776.9	0.170	–

BNT/BNT/BNT: three doses of BNT162b2. ChAd/ChAd/BNT: two doses of ChAdOx1 nCoV-19 and booster dose of BNT 8. ChAd/BNT/BNT: first dose of ChAdOx1 nCoV-19, second and booster doses BNT162b2. *Mann–Whitney test

Four weeks after the second dose the IgG levels (475.7 AU/mL) of the ChAd/BNT (193.4–873.3) group were statistically significantly higher than the ones of control group BNT/BNT, 111.8 AU/mL (63.2–151.3), and ChAd/ChAd, 50.16AU/mL (11.95–94.4) (*Mann–Whitney test*).

Among the tested cohort, the percentage of participants reaching an IgG anti-S-RBD titer over 100 AU/mL was 55% in BNT/BNT, 85% in ChAd/BNT, and only 25% in ChAd/ChAd (Table 2).

The neutralizing antibody titers a month after the second injection were significantly higher in the ChAd/BNT group than in patients treated with ChAd/ChAd or BNT/BNT (*p* < 0.05) (Fig. 1).

**Fig. 2:**
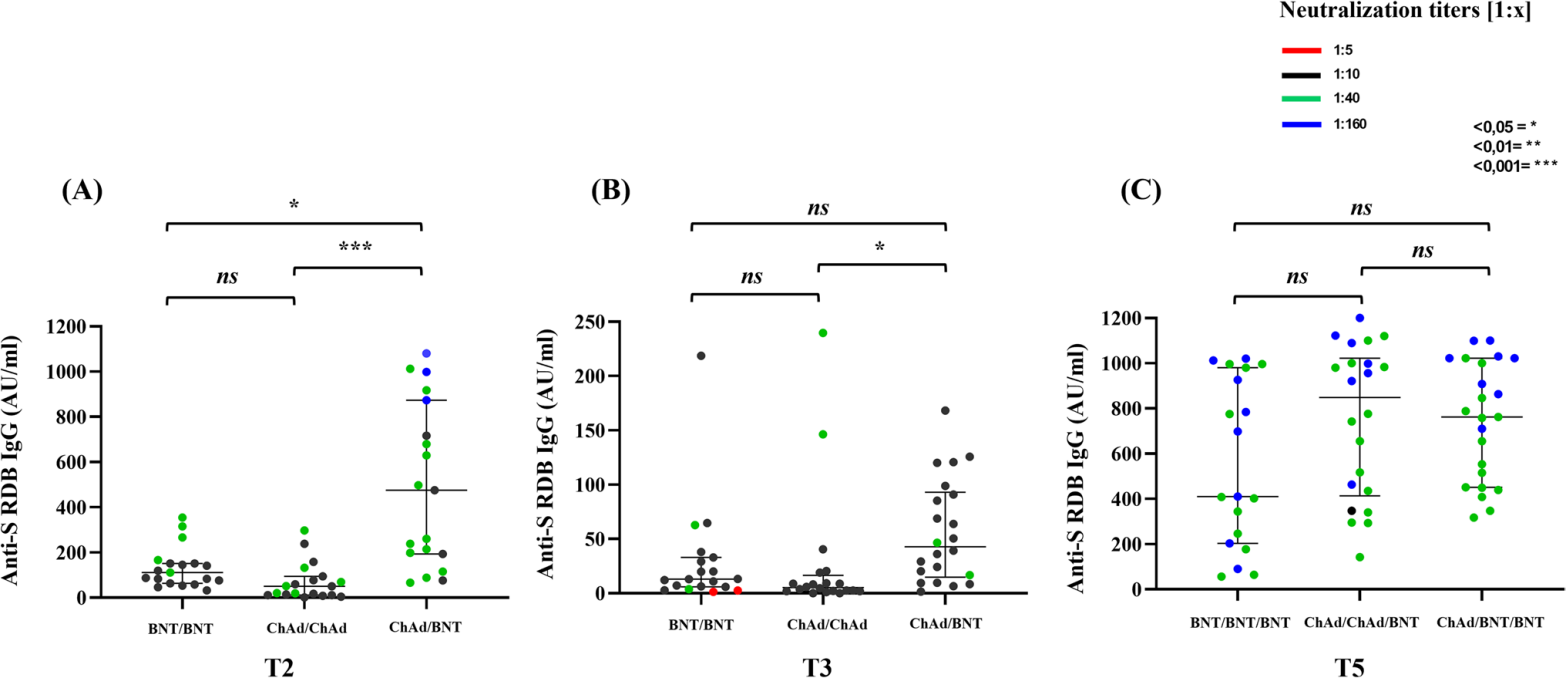
Distribution of IgG anti-S-RBD antibody and neutralization titers according to the vaccination schedule: ChAdOx1 nCoV-19 as prime and BNT162b2 mRNA as a booster (ChAd/BNT), or homologous ChAdOx1 nCoV-19 (ChAd/ChAd) or BNT162b2 (BNT/BNT). Sera from ChAd/BNT (23), ChAd/ChAd (23), or BNT/BNT (20) were evaluated for IgG anti-S-RBD antibody and neutralization titers at different time points as indicated (A-C). T2 (4 weeks) and T3 (20 weeks) and 4 weeks after the end of the vaccination regimen (T5)

No significant difference was detected in neutralizing antibody titers of subjects treated with ChAd/ChAd and the control group (BNT/BNT) (Fig. 2, panel A).

### Anti-S-RBD antibody levels and neutralization titers at T3 (20 weeks) after the second dose

At T3, 20 weeks after the second dose, the median IgG anti-RBD titer was reported as 13 AU/mL (5.84–33.07) in the BNT/BNT control group, 42.91 AU/mL (14,96–92,98) in the ChAd/BNT group, and 34AU/mL (2.05–16,55) in the ChAd/ChAd group. Moreover, the ChAd/BNT group showed a significant difference in comparison with the ChAd/ChAd regimen group (*p* = 0.003).

As reported in Fig. 2, panel B, we have measured the capability of our cohort’s serum samples to neutralize SARS-COV-2 viral particles 20 weeks after the second dose, and a significant difference was reported only between the ChAd/BNT and the ChAd/ChAd groups. (*p* < 0.05).

### Anti-S-RBD antibody levels and neutralization titers at T5, 4 weeks after the third dose against wt and BA.1 variant

Our cohort of vaccinated subjects was sampled after receiving the BNT162b2 mRNA booster (1 month after the third dose) and results have shown an increase in the antibody titer to a median value of 775.3 AU/mL (450.7–1022) in the group, 849 AU/mL (413.7–1022) in the homologous ChAd/ChAd /BNT and 410.5 AU/mL (203–980) in the control group.

The assay for IgG to RBD with Maglumi showed that all subjects that had received BNT162b2 as a third vaccine dose developed a comparable positive antibody response, which resulted slightly lower in the ChAd/ChAd group.

In this cohort, no statistical difference in neutralizing antibody titers to *wt* was reported after the third injection of the BNT162b2 booster, regardless of the type of vaccine used (Fig. 2, panel C).

**Fig. 3:**
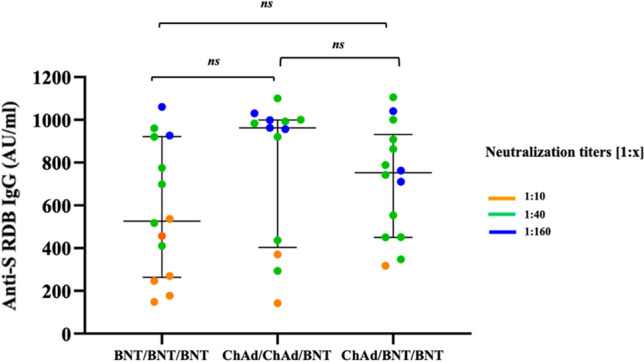
IgG anti-S-RBD antibody and neutralization titers according to the vaccination schedule: ChAdOx1 nCoV-19 as prime and BNT162b2 mRNA as a booster (ChAd/BNT), or homologous ChAdOx1 nCoV-19 (ChAd/ChAd) or BNT162b2 (BNT/BNT). Sera from ChAd/BNT (14), ChAd/ChAd (13), or BNT/BNT (14) were evaluated for IgG anti-S-RBD antibody and neutralization titers against Omicron BA.1 variant, 4 weeks after the BNT162b2 booster dose

Importantly, the neutralization competence against the Omicron BA.1 variant (*n* = *40*) after the completion of the vaccination schedule was studied in a subgroup (*n* = *40*) of subjects included in our cohort and treated with the three vaccine regimens. Our data show that 100% of participants are simultaneously positive for neutralizing antibodies against the SARS-COV-2 *wt* strain and Omicron BA.1 variant. Moreover, none of the samples have shown results of negative neutralization titers against the Omicron BA.1 variant. Altogether, these results indicate that the booster dose has increased neutralizing antibodies in all treated against the SARS-COV-2 *wt* strain and has highlighted that Omicron BA.1 was cross-neutralized at comparable levels Fig. 3.

The rate of responders against the Omicron BA.1 variant was 100% in all analyzed g.

### Breakthrough infections and neutralization titers against SARS-CoV-2 wt and Omicron BA.1 strain

Among the CORIMUN study cohort, we have also analyzed a subgroup of subjects (16 included in the BNT/BNT group and 14 belonging to the homologous ChAd/ChAd and heterologous ChAd/BNT groups) that had tested positive for SARS-CoV-2 after the BNT162b2 third dose. The sera of these participants were assayed for neutralization titers against SARS-CoV-2 *wt* and Omicron BA.1 variant. All the samples resulted in positive neutralization titers (results ≥ the established cut-off value of 1:10). The homologous BNT-BNT-BNT group had the most robust increase of neutralizing titers (31%, (5/16) developed 1:640 neutralization titer; 31% (5/16), 1:160; 12,5% (2/16), 1:40 and 25% (4/16),1:10) against the original SARS-COV-2 *wt* strain compared to the homologous and heterologous group, where none of the tested subjects developed such high titers (43% (6/14), 1:160; 43% (6/14), 1:40, and 14% (2/14) 1:10). However, no increase in neutralizing titers was detected when sera from BNT/BNT/BNT group were tested against the Omicron BA.1 variant.

**Fig. 4:**
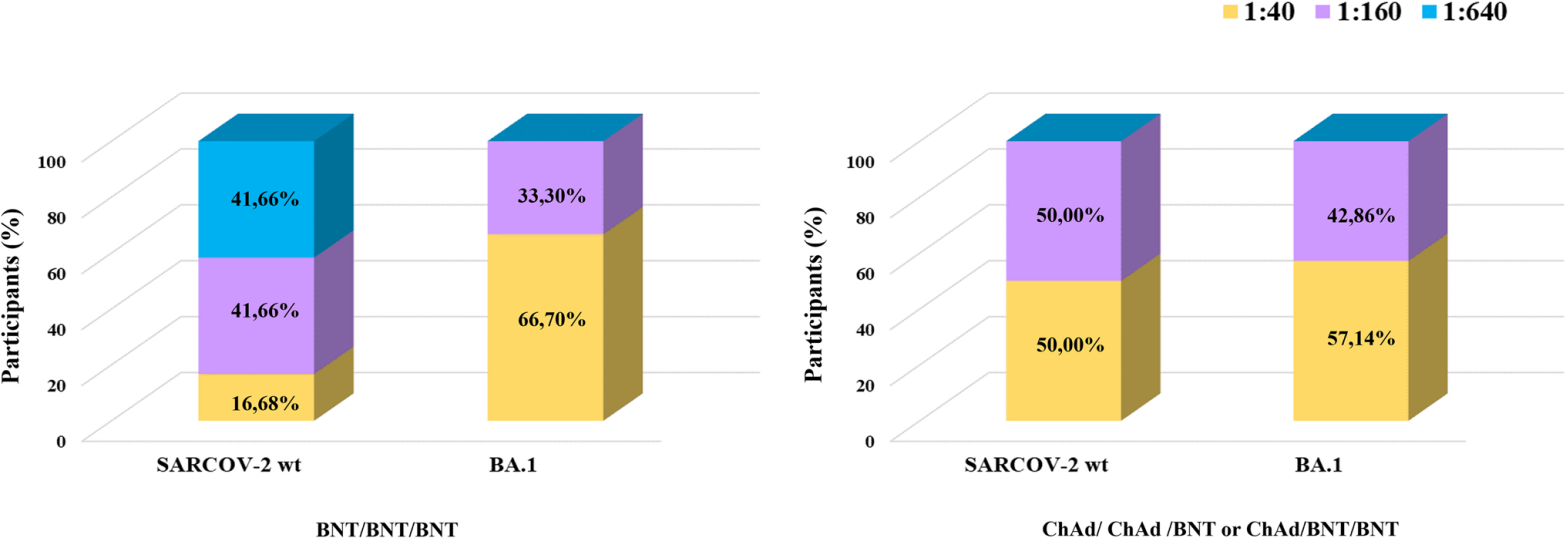
Rate of subjects (%) with a neutralizing antibody titer over the established cut-off (1:10) against the SARS-COV-2, *wt* strain, and Omicron BA.1 variant

In accordance with previous reports, after natural infection, the neutralization titers of the BNT/BNT/BNT group showed a notable drop against the BA.1 variant when compared to that determined against *the wt* SARS-COV-2 strain (Fig. 4).

The neutralization titers after natural infection in subjects from the ChAd/BNT/BNT and ChAd/ChAd/BNT groups showed a comparable neutralization activity against *wt* and BA.1 strains, and no reduction was detected against the Omicron BA.1 strain (Fig. 4).

## Discussion

Within the CORIMUN study cohort, our study aimed to analyze the humoral response in terms of antibody response to SARS-CoV-2 spike protein and neutralizing activities of a real-world cohort of healthy Sardinian subjects (healthcare workers, post-graduate medical trainees, and researchers at the University of Cagliari), who have been vaccinated using the heterologous priming with ChAdOx1 (ChAd) vector vaccine, followed by a BNT162b2 mRNA booster dose (ChAd/BNT/BNT) compared to homologous dosing (ChAd/ChAd/BNT) and BNT/BNT/BNT employed as a “reference” group.

This real-world data showed that, after the second dose, anti–SARS-CoV-2 S-RBD IgG titers were higher in the group of participants who had received the heterologous viral vector and mRNA vaccines (ChAd/BNT) followed by homologous mRNA vaccine (BNT/BNT group) and homologous viral vector. After the rare adverse event associated with adenovirus-based vaccines, heterologous immunization has focused a lot of attention on itself.

As a matter of fact, our results reinforce the findings of other studies [14, 15] that report higher antibody responses after mRNA vaccination in subjects who had received ChAd as their first dose. Barros-Martins and coauthors [16] have described a significantly larger antibody response against the SARS-CoV-2 spike protein for ChAd/BNT than for ChAd/ChAd, and they have suggested that heterologous vaccines prove greater effectiveness in stimulating broader immunity [17].

As expected, at T3, 20 weeks after the second dose, the IgG levels had all decreased (10 times).

After the booster dose, homologous regimens have induced antibody responses similar to those of heterologous administrations. Decreasing immune responses were effectively restored after a 3rd BNT vaccination [18, 19]. Accordingly, all study participants among our cohort have developed high and similar neutralizing titers after completing the vaccination regimen.

Several studies have previously described [14, 20, 21] that ChAdOx/mRNA regimens led to a strong induction of antibodies.

We have described immune responses at 4 and 20 weeks after the second dose and included the follow-up after the third dose; our research could therefore be considered as an integration of other current publications, as it provides additional reassurance about effective antibody responses after heterologous vaccination treatments.

The emergence of variants has caused concern about the effectiveness of immunity after vaccination [22, 23]. For example, the Omicron variant was discovered in South Africa during the booster vaccination campaigns.

This emergence was related to an increase in hospitalization cases and infection severity. A high number of mutations harbor in the S proteins of the Omicron variants BA.1, which increases immune evasion and the potential for transmissibility [24–26].

The neutralization competence against the Omicron BA.1 variant was analyzed among a subgroup of our cohort after the completion of the vaccination schedule.

At the latest time point measured, 30 days after the BNT162b2 third dose, all tested sera (40) retained neutralizing activity against the currently dominant Omicron BA.1 variant, regardless of the type of vaccination regimen [27]. The BNT162b2 booster’ in particular induced titers that were neutralizing BA.1 almost as potently as SARS-COV-2 *wt*. This finding has highlighted that Omicron BA.1 was cross-neutralized at comparable levels in those with hybrid immunity and treated with a heterologous vaccine schedule. Despite Omicron's evasive immune properties, our findings are in line with other reports that show that a third dose of mRNA vaccination generates higher responses against Omicron [18, 28–30].

These data suggest that vaccines based on the ancestral Wuhan strain are likely able to induce cross-reactive antibodies and protect against this variant.

When neutralizing antibodies induced via natural infection after vaccination regimens were compared, we observed in BNT homologous vaccinated higher neutralization titer against the ancestral virus than the BA.1 variant. For individuals who had received the heterologous vaccination, neutralization activity against wt and BA.1 resulted equivalent.

This could suggest an enhancement of the anti-SARS-CoV-2 immune response, potentially caused by re-exposure, stronger in individuals subjected to heterologous vaccination.

We are aware that our current study has several limitations. Firstly, the studied cohort is small when compared to other clinical cohorts and the obtained results may therefore have been limited by such limited selection. Secondly, the recruited participants may not be representative of the general population, as our cohort included healthcare workers and subjects with no history of comorbidities, which may have narrowed the range to subsections that were healthy or at low risk of infection. Lastly, only the humoral immune response was analyzed in our work and T cell (cellular) responses to vaccination were not investigated.

Nevertheless, despite the limited number of clinical specimens, we were able to test the neutralization activity, which remains the main correlate of protection, against the variant of concern Omicron BA.1, which was arising during our study.

This important acquisition shows how current variants can influence the effectiveness of vaccine regimens and how neutralization titers may be related to protection. Furthermore, the 90% neutralization titer endpoint employed in our live SARS-CoV-2 virus neutralization assays is more rigorous than the 50% commonly described in most published literature.

## Conclusions

These data may be of interest and provide additional support in case of concerns regarding vaccine supply. Further assessments on the magnitude of the immune response after the fourth dose are needed. They are crucial for the update of future vaccine strategies.

## Data Availability

Data and materials are available from corresponding authors upon reasonable request.
